# Evanescent extraosseous calcifications in low turnover bone: management and outcomes: a case report

**DOI:** 10.3389/fneph.2025.1702475

**Published:** 2026-01-14

**Authors:** Mariel Hernandez-Pérez, Daniel Enos

**Affiliations:** Department of Nephrology, Healthcare Complex Dr. Victor Rios Ruiz (CAVRR), Los Angeles, Biobio Province, Chile

**Keywords:** low bone turnover, extraosseous calcifications, sodium thiosulphate, adynamic bone disease, peritoneal dialysis

## Abstract

**Introduction:**

The prevalence of bone disease in peritoneal dialysis patients has been recently shown to exceed 54%, including patients with parathormone (PTH) levels within the theoretical adequate target, yet demonstrating low bone turnover on histomorphometry. Moreover, bone disease is often associated with abnormalities in calcium and phosphate metabolism, leading to tissular deposits such as extraosseous calcifications.

**Case presentation:**

We present a 22-year-old female patient managed on peritoneal dialysis with persistent swelling of all four extremities, including the fingers, hands, and feet, accompanied by a marked decrease in PTH. Many extraosseous calcifications in the hands were seen in the X-ray images, prompting a switch from peritoneal dialysis to conventional high-flow haemodialysis and intravenous sodium thiosulphate (STS) therapy. The patient showed adequate treatment tolerance, with most calcifications disappearing after 3 months of therapy.

**Conclusions:**

Our experience suggests that the treatment of extraosseous calcifications requires timely and multi-angle intervention. At the same time, treatment with STS has proven effective and well tolerated in this patient.

## Introduction

Mineral and bone disorders (MBD) are common complications of chronic kidney disease (CKD) associated with cardiovascular outcomes and mortality in dialysis patients (1–5). The spectrum of bone disease in peritoneal dialysis patients is not yet fully understood. It has been recently shown that up to 54% of patients with parathormone (PTH) within the goal recommended by Kidney Disease Improving Global Outcomes (KDIGO) (6) exhibit low bone turnover on histomorphometry, according to Pereira et al. (7). In this case, many abnormalities of calcium and phosphate metabolism are described, mainly, the presence of positive calcium balance, which may result in PTH over-suppression (8, 9). In addition, peritoneal dialysis patients frequently have positive phosphorus balance, reflecting an ongoing intrinsic inflammatory activity, which can lead to extraosseous calcifications, especially vascular calcifications, regardless of bone turnover (10). When serum calcium is reduced, controlling phosphate and parathyroid levels is important to prevent extraosseous calcifications. Sodium thiosulphate (STS) has been used for managing metastatic calcifications, acting through three complementary mechanisms, namely, as a potent serum calcium chelator, an antioxidant, and a vasodilator agent. Herein, we report a case of a young female patient on peritoneal dialysis with adynamic bone disease (ABD) and extraosseous calcifications, successfully treated with intravenous STS as the cornerstone therapy.

## Case presentation

A 22-year-old female patient with severe lupus nephritis and end-stage renal disease undergoing immunosuppressive treatment with prednisone, mycophenolate mofetil, and hydroxychloroquine, was managed on continuous ambulatory peritoneal dialysis (PD) for almost 2 years. She was receiving the following dialysis prescription: total time: 10-h/dwelling period: 2 h/concentration: dextrose 2.5%/inflow: 1.8 L.

**Figure 1:**
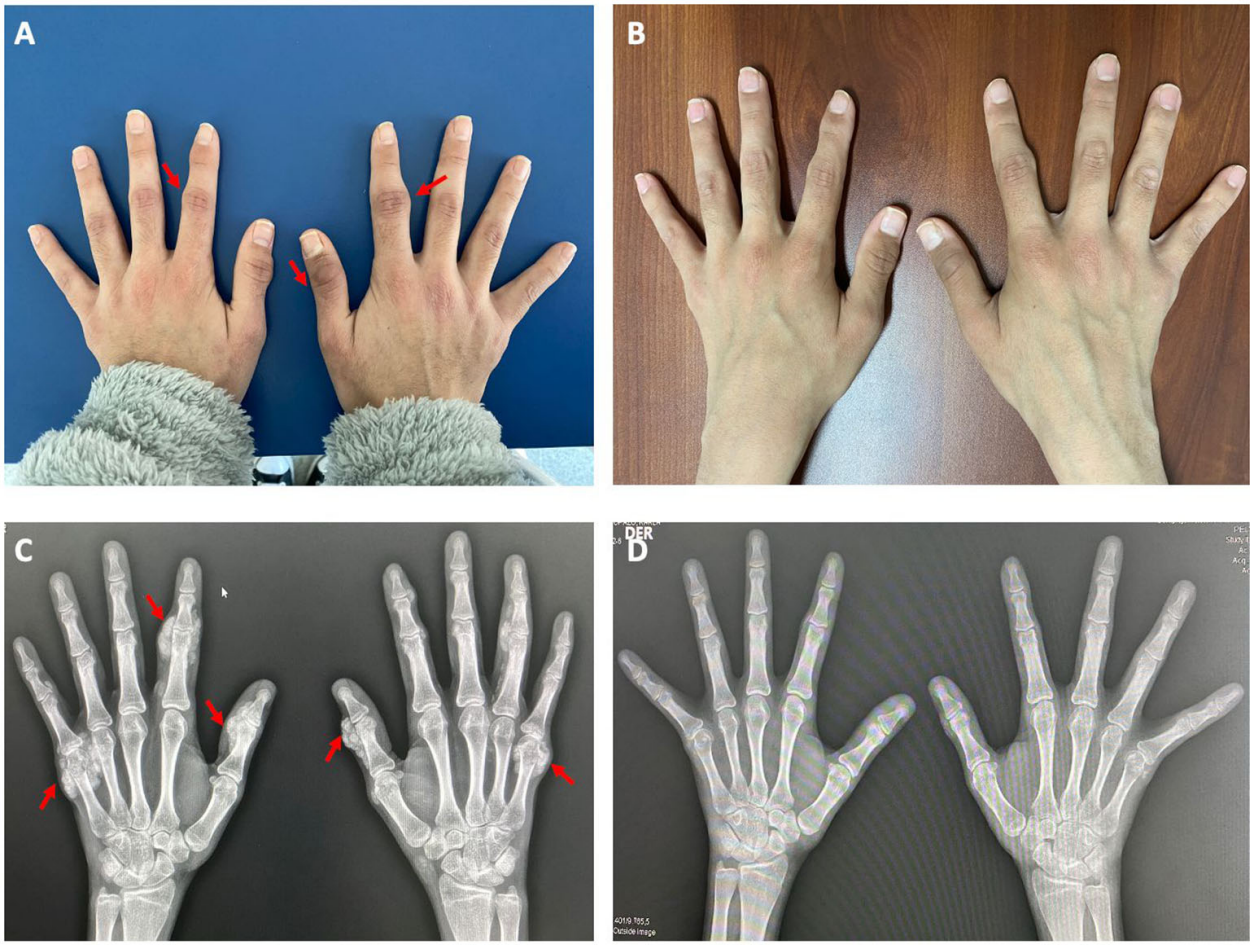
Extraosseous calcifications (amorphous and multilobulated periarticular calcifications) at baseline and 3 months after treatment. **(A)** and **(C)** show the photograph of extraosseous calcifications in fingers and hands and the X-ray at baseline, respectively. **(B)** and **(D)** show the photograph of both hands and the X-ray without extraosseous calcifications, 3 months after the start of treatment.

During the first year, PTH levels were ​​between 247 and 430 pg/mL while using calcimimetics. After 2 years of starting dialysis, her PTH levels fell below 200 pg/mL, prompting the withdrawal of the calcimimetic. After 6 months of discontinuation, she developed painful and persistent swelling of the small joints, encompassing multiple fingers, hands, and feet. At the same time, she had a high calcium–phosphorus product and severe hyperphosphemia despite high doses of calcium chelators. Hospitalisation was decided for a diagnostic workup, which revealed serum calcium of 8.1 mg/dL, phosphorus 8.5 mg/dL, alkaline phosphatase 65 UI/L, 25-hydroxyvitamin D 13.7 ng/mL, C-reactive protein 7 mg/dL, and PTH 47 pg/mL. Radiographs demonstrated amorphous and multilobulated periarticular calcifications in the proximal phalanges, metacarpals, and metatarsals (**Figure 1**).

We initiated immediate therapeutic interventions, switching the patient to conventional high-flow haemodialysis, prescribed three times a week, 5-h sessions, and pharmacological management: cinacalcet was withheld, calcitriol was discontinued, phosphorus chelator was changed from calcium acetate to sevelamer carbonate, and finally, STS 25 g was administered intravenously after each haemodialysis session. Furthermore, we performed a bone biopsy from the anterior superior iliac crest, following the technique described by Barreto et al. (11), preceded by a pre-labelling procedure (12). Histomorphometry analysis confirmed ABD, with the following parameters: bone formation rate (BFR/BS) 0.0004 mm^3^/mm^2^/day, osteoid volume (OV/BV) at 1%, and bone marrow fibrosis (FB) 0%.

The patient showed adequate treatment tolerance, with the calcifications disappearing after a 3-month period of full therapy (**Figure 1**), and although phosphorus levels were not controlled, the other parameters were in adequate values, such as serum calcium of 9.4 mg/dL, phosphorus 8.5 mg/dL, alkaline phosphatase 85 UI/L, and PTH 111 pg/mL.

After 9 months of STS therapy, she was transferred to a dialysis centre near her home, where session time was reduced to 4 h, achieving better control of calcium and phosphorus (**Table 1**). Subsequently, the patient continued under a close follow-up in our outpatient clinic, remaining free of new lesions 1 year after STS was suspended, reaching adequate management of calcium and phosphorus although the PTH level was above the standard goal, requiring calcimimetics to be restarted, 1 year after the initial diagnosis (**Table 1**).

**Table 1:** Mineral metabolism parameters and treatment during follow-up.

Date follow up	June 2022	July 2022	October 2022	April 2023	July 2023	April 2024
Laboratories						
PTH (pg/mL)	47	55	111	98	600	345
AP (UI/L)	65	81	85	159	159	174
Ca (mg/dL)	8.1	9.2	9.4	9.6	8.3	9.4
P (mg/dL)	8.6	9.7	8.5	5	4.8	4.7
Ferritin (ng/mL)	640		381		570	425
Therapeutic strategies						
Chelator of P	Calcium acetate	Sevelamer	Sevelamer	Sevelamer	Sevelamer	Sevelamer
Sodium thiosulphate	No	Yes	Yes	No	No	No
Renal replacement therapy	DPAC	HD	HD	HD	HD	HD
Dialysis time (h)		5	5	4	4	4

PTH, parathormone; AP, alkaline phosphatase; Ca, calcium; P, phosphorus; DPAC, continuous ambulatory peritoneal dialysis; and HD, haemodialysis.

## Discussion

We present the case of a young patient after 2 years on PD with low turnover bone disease, complicated by extraosseous calcification. After describing the clinical presentation, we list the available therapeutic strategies to achieve progressive regression of the lesions.

It was essential to consider the possible differential diagnoses for this type of periosteal lesions, since they could be confused with metastatic cancer, bone cysts, and osteosarcoma. Although histologic confirmation is unavailable, findings such as serum PTH, calcium, and phosphorus levels, in a CKD patient context, play a fundamental role in diagnostic certainty. A complete evaluation of medical history, biochemical, and radiographic findings may be sufficient to achieve a correct diagnosis and guide appropriate treatment management.

### Pathophysiology of calcification

It is well known that extraosseous calcifications are a rare complication of CKD–MBD and are usually more common in poor and developing countries associated with the massive use of calcium-based phosphate binders and calcium-rich dialysis concentrates. It starts primarily with the supersaturation of plasmatic calcium and phosphorus. In addition, PD may exacerbate this pathologic mechanism, as these patients tend to have positive phosphorus balance unless they are severely malnourished (13).

Furthermore, it is well known that the primary means to improve phosphate transfer across the peritoneal membrane is almost exclusively diffusive process, needing to increase the number of daily peritoneal dialysis cycles and the dwell time of the dialysis solution (14). The use of calcium phosphate binders represents another risk factor because it can induce positive calcium balance that could lead to PTH over-suppression, favouring the development of ABD (13, 15).

In the present case, it was clear that CKD-MBD initially presented with PTH levels within the expected range, even with the use of calcimimetics. However, later during follow-up, it was shown that PTH over-suppression failed to recover despite more than 6 months after discontinuation of cinacalcet, resulting in a biochemical pattern of low bone turnover confirmed on biopsy.

### Role of dialysis prescription and phosphate binders

Although the mechanisms underlying the development of ABD are complex, there are two important aspects that should be emphasised. First, overtreatment of secondary hyperparathyroidism without appropriate follow-up could be a key factor contributing to the increase in ABD diagnoses. Second, there is likely a genuine need to redefine the PTH target values. In line with this concept, low bone turnover should be considered when the PTH level is below 150 pg/mL in patients with advanced CKD, with a sensitivity and specificity for diagnosis of 68.6% and 61.2%, respectively (16, 17). However, the target PTH range proposed for CKD patients on dialysis (6) is not a guarantee of normal bone turnover, as illustrated by our case. Pereira et al. analysed 49 patients with CKD on peritoneal dialysis who underwent bone biopsy and found ABD to be a common pattern (42.9%) in patients with PTH within the guideline-recommended plasmatic range (6). Overall, ABD was found in 59% of cases, with a median PTH of 312 (60–631) pg/mL in patients with adynamic bone (7).

Moreover, identifying the underlying causes of low bone turnover is a decisive step in guiding therapeutic strategies, as ABD management is usually multitargeted. This includes a change of dialysis therapy with better control of hypercalcemia and hyperphosphatemia, using calcium-free phosphate binders, and maintaining neutral calcium balance with no net flux of calcium from the bones to the extracellular fluid. Theoretically, with a dialysate calcium concentration of 2.5 mEq/L, net flow of calcium should not occur (18). Although the 2007 CKD–MBD guidelines suggest the use of a calcium concentration in the dialysate between 2.5 and 3.0 mEq/L, a recent kinetic modelling study in haemodialysis patients (19) depicted that a dialysate calcium concentration less than 2.5 mEq/L would be necessary to prevent long-term calcium accumulation in a significant proportion of patients, allowing a small amount of calcium to be removed during ultrafiltration.

Regarding the effect of calcium in the dialysate on bone turnover, it is well known that patients treated with a dialysate calcium concentration of 2.5 mEq/L show changes in parameters, indicative of higher bone turnover. This is likely due to prevention of positive calcium balance and sustained stimulation of PTH secretion, thereby helping to prevent low turnover (20, 21). 

In addition, the use of sevelamer has been extensively studied, achieving better control of phosphemia without concomitant use of elemental calcium, eliminating the risk of additional calcium absorption. Furthermore, experimental studies in murine models using sevelamer have shown that apart from normalising serum phosphorus, surprisingly, sevelamer can reverse CKD-induced trabecular osteopenia, increasing osteoblast surfaces in the metaphyseal trabeculae of the tibia and femur, and reinforcing osteoid surfaces, demonstrating remarkably higher bone formation rates (22). Subsequently, Ferreira et al. compared patients who underwent bone biopsies at the beginning and end of a 1-year-period while receiving sevelamer hydrochloride or calcium carbonate. No statistically significant differences in bone turnover or mineralisation were observed between the groups; however, bone formation rate was higher and trabecular architecture improved only in the sevelamer group (23, 24).

### Sodium thiosulphate therapy

STS is a well-known drug, with limited but growing evidence, especially from the last decade, mostly in calciphylaxis management (25). Despite several mechanisms proposed, involving complexation with calcium ions and dissolution of calcium deposits, these were recently challenged by O’Neill and Hardcastle (26), who proposed a more relevant antioxidant action that targets inflammation and intimal hyperplasia based on the ability of thiosulphate to be oxidised to sulphate.

Additionally, thiosulphate can be generated in tissues from the mitochondrial sulphide oxidation pathway, being a stable, nontoxic metabolite, and could alter the activity of proteins from many cellular signalling pathways involved in apoptosis, angiogenesis, inflammation, metabolism, proliferation, and oxygen sensing. It can also play a detoxifying role during oxidative stress by increasing the development of glutathione (27).

Moreover, only a few case reports have demonstrated the efficacy of STS in the treatment of extraosseous calcification (28–31). Although there is no strong evidence, STS prescription remains dependent on availability, tolerance, and safety. Its effects appear to achieve partial regression, possibly due to low STS doses at the beginning of therapy, in an effort to avoid adverse reactions.

Our patient was started on full dose, and continued without interruptions, as it was well tolerated. X-ray images promptly demonstrated a substantial reduction in the size of extraosseous calcifications, accompanied by a significant symptomatic improvement. We believe that this more aggressive therapeutic approach facilitated faster lesion regression, needing only a shorter period of treatment for near-complete resolution, without residual lesions after more than 1 year of follow-up after treatment was discontinued.

Finally, it is important to emphasise that CKD–MBD patterns change over time, and the incidence of ABD is increasing, making it necessary, in our opinion, to redefine PTH cutoff points, mainly in PD patients. Likewise, close monitoring of biochemical parameters that guide variations in turnover is necessary to prevent potential risk factors related to low bone turnover, such as age, uraemia, calcium overload, excessive PTH suppression resulting from vitamin D receptor activators, PD itself, and diabetes mellitus.

Key take-away lessons include the importance of early detection and a combined therapeutic approach. Strategies such as the use of non-calcium chelating agents, the change of dialysis therapy or adjustment of dialysis prescription, and the use sodium thiosulphate, represent the most effective means to achieve better outcomes.

## Data Availability

The raw data supporting the conclusions of this article will be made available by the authors, without undue reservation.
